# Diagnosis of “intensive care unit-acquired weakness” and “critical illness myopathy”: Do the diagnostic criteria need to be revised?

**DOI:** 10.1016/j.cnp.2024.08.002

**Published:** 2024-08-14

**Authors:** Belén Rodriguez, Joerg C. Schefold, Werner J. Z’Graggen

**Affiliations:** a Department of Neurosurgery, Inselspital, Bern University Hospital, University of Bern, Bern, Switzerland; b Department of Intensive Care Medicine, Inselspital, Bern University Hospital, University of Bern, Bern, Switzerland; c Department of Neurology, Inselspital, Bern University Hospital, University of Bern, Bern, Switzerland

**Keywords:** Critical illness, Muscular dysfunction, Muscle weakness, Electrophysiology, Outcome, Mortality

## Abstract

**Objectives:**

Intensive care unit acquired weakness (ICUAW) is a clinical diagnosis and an umbrella term for acquired weakness due to neuromuscular disorders such as critical illness myopathy (CIM) but also muscular inactivity/atrophy. Without a clear understanding of the distinct aetiology, it seems difficult to predict outcomes of ICUAW and to test and apply effective future treatments. The present study contrasts ICUAW with CIM and assesses the diagnostic and clinical relevance for affected patients.

**Methods:**

Data from a previous prospective cohort study investigating critically ill COVID-19 patients was analysed in a retrospective fashion. Patients were examined ten days after intubation with clinical assessment, nerve conduction studies, electromyography and muscle biopsy. Mortality was assessed during critical illness and at three months after hospital discharge. ICUAW and CIM were diagnosed according to the current diagnostic guidelines.

**Results:**

In this patient sample (n = 22), 92 % developed ICUAW, 55 % developed ICUAW and CIM, and 36 % had ICUAW but did not develop CIM. Overall, 27 % patients died during their stay in the intensive care unit. At three months after discharge, there were no further deaths, but in 14 % of patients the outcome was unknown. The diagnosis of CIM was more strongly associated with death during critical illness than ICUAW. No patient with ICUAW who did not fulfil the criteria for CIM died. Both clinical and electrophysiological criteria showed excellent sensitivity for CIM diagnosis, but only electrophysiological criteria had a high specificity. Determination of the myosin:actin ratio showed neither high sensitivity nor specificity for the diagnosis of CIM.

**Conclusions:**

The results of the present study support that ICUAW is a non-specific clinical diagnosis of low predictive power with regard to mortality. Further, diagnosing “ICUAW” seems also of little research value for both exploring the aetiology and pathophysiology of muscle weakness in critically ill patients and for evaluating potential treatment effects. Thus, more specific diagnoses such as CIM are more appropriate. Within the different diagnostic criteria for CIM, electrophysiological studies are the most sensitive and specific examinations compared to clinical and muscle tissue assessment.

**Significance:**

Avoiding an overarching diagnosis of “ICUAW” and instead focusing on specific diagnoses appears to have several relevant consequences: more precise diagnosis making, more accurate referral to aetiology and pathophysiology, improved outcome prediction, and development of more appropriate treatments.

## Highlights

• In this patient cohort, 92 % met the criteria for ICUAW, only 55 % met those for CIM, and 36 % had ICUAW but did not develop CIM.
• CIM was more strongly associated with death during critical illness than ICUAW; no patient with ICUAW but without CIM died.
• Electrophysiological studies have a high sensitivity and specificity for CIM diagnosis.

## 1 Introduction

Weakness as a complication of critical illness and intensive care treatment is very common; particularly during the COVID-19 pandemic, this has been an issue of great concern. Many COVID-19 patients required prolonged intensive care, partly due to the development of weakness, resulting in a shortening of available hospital beds and a high occupancy of rehabilitation facilities, and ultimately leading to ongoing challenges for the health care system (Cabanes-Martinez et al., 2020, Frithiof et al., 2021, Rodriguez et al., 2022). Generalized weakness and muscle atrophy in critically ill patients usually occur symmetrically and primarily affect the proximal limbs and respiratory muscles (Latronico and Bolton, 2011, Schefold et al., 2020). This phenomenon is increasingly referred to as “intensive care unit-acquired weakness” (ICUAW). The diagnosis of ICUAW requires the assessment of muscle strength of 12 muscle groups using the Medical Research Council (MRC) score. A sum score of less than 48 is required for diagnosis (De Jonghe et al., 2002, Schefold et al., 2020). The reported incidence of ICUAW varies widely depending on the length of stay in the intensive care unit (ICU) and mechanical ventilation in particular, and between the study centres, ranging from 25 to 75 % (Ali et al., 2008, Bercker et al., 2005, Hermans et al., 2014, Mirzakhani et al., 2013, Nanas et al., 2008, Sharshar et al., 2009). Patients experiencing weakness during critical illness generally face worse outcomes and have an elevated risk of mortality within the first year post-ICU discharge (Hermans et al., 2014, Herridge et al., 2016, Herridge et al., 2011). While recovery typically occurs over several months, some patients may continue to experience weakness for an extended period, possibly persisting for years (Hermans et al., 2014, Kamdar et al., 2017).

ICUAW serves as a clinical diagnosis and is an umbrella term for weakness due to muscular inactivity atrophy and several acquired neuromuscular disorders, and thus encompasses various neuropathological entities (Howard et al., 2008). Among the most prevalent causes of neuromuscular weakness during critical illness are critical illness myopathy (CIM), critical illness polyneuropathy and the combined occurrence of these two conditions. Other potential causes include Guillain-Barré syndrome, myasthenia gravis, other acute myopathies including myositis and neuropathies, drug overdose, and other conditions that lead to acute neuromuscular weakness. These different disorders have different underlying aetiologies and pathophysiologies, therefore require distinct preventive and therapeutic measures, and often also have a varying prognosis (Latronico and Bolton, 2011, Schefold et al., 2020). Differential diagnosis requires laboratory investigations, electrophysiological examinations, and in some cases imaging and/or a muscle biopsy. For example, in CIM, which is characterized as an acute acquired primary myopathy, diagnostic criteria are based on a comprehensive approach involving the presence of critical illness, clinical signs of weakness, electrophysiological signs of myopathy, and evidence of primary myopathy with myosin loss (Latronico and Bolton, 2011).

Without a clear understanding of the underlying causes, it is difficult to predict the outcomes of acquired weakness during critical illness and to administer effective treatment. Therefore, the use of ICUAW as an umbrella diagnosis is not suitable for both clinical practice and research purposes. The present study addresses this issue as a first step by contrasting the diagnosis of ICUAW with that of CIM and assessing their diagnostic and clinical relevance for patients and their outcomes.

## 2 Methods

### 2.1 Study design and participants

In this study, a sub-dataset from a previous prospective cohort study investigating COVID-19 patients is retrospectively analysed (Rodriguez et al., 2022). The prospective study included 31 COVID-19 patients with acute respiratory distress syndrome admitted to the ICU of the University Hospital Bern, Switzerland, who required mechanical ventilation (registration-URL: clinicaltrials.gov; unique identifier: NCT04397172), were aged between 18 and 80 years, and examined between April and December 2020. Exclusion criteria were pre-existing intubation for more than 24 h, pregnancy, breastfeeding, and pre-existing polyneuropathy, Guillain-Barré syndrome, spinal cord lesion, myasthenia gravis, or myopathy. All procedures were approved by the local ethics committee (Kantonale Ethikkomission, Bern, Switzerland: project-ID 2020-00730) and complied with the Declaration of Helsinki and its amendments. Patients who met the eligibility criteria were included if written informed consent was obtained from an independent physician acting as a proxy. Next-of-kin were informed as soon as possible, and written informed consent was obtained as soon as the patient's health status permitted.

Patients were examined within 24 h, and followed up after two, five and ten days after intubation. In the present retrospective study, only data from the last examination day was analysed. This sub-dataset includes data from clinical examinations, nerve conduction studies, electromyography (EMG) and muscle biopsy.

### 2.2 Procedures

#### 2.2.1 Clinical examination

The muscle strength sum score was determined by summarizing the muscle force measurements using the MRC scale for three functional muscle groups in both the upper (shoulder abduction, elbow flexion, and wrist extension) and lower extremities (hip flexion, knee extension, and ankle dorsiflexion) (Schefold et al., 2020).

#### 2.2.2 Electrophysiological studies

Standard motor and sensory nerve conduction studies were performed according to conventional protocols. The examination focused on one nerve each in the upper and lower extremities. Nerve conduction studies were performed in the upper extremities from the median or ulnar nerve depending on accessibility (e.g. arterial or venous catheters), and in the lower extremities from the peroneal nerve. The measured parameters for motor nerve conduction studies were distal motor latencies, nerve conduction velocities, and amplitudes of the compound muscle action potentials, and for sensory nerve conduction studies, nerve conduction velocities and amplitudes of sensory action potentials. For motor nerve conduction studies, the median nerve was stimulated at the wrist and at the cubital fossa. The ulnar nerve was stimulated at the wrist and proximal to the ulnar sulcus. Recordings were made from the abductor pollicis brevis or the abductor digiti minimi muscle. The peroneal nerve was stimulated distal from the fibular head and in the fossea poplitea. Recordings were made from the tibialis anterior muscle. Repetitive motor nerve stimulation, targeting either the median or ulnar nerve was used to detect neuromuscular junction dysfunction. Repetitive stimulation at 3 Hz (10 stimuli) was recorded from the abductor pollicis brevis or the abductor digiti minimi muscle. Sensory nerve conduction studies were performed from the median or ulnar nerve and from the sural nerve using surface electrodes. Needle EMG of the brachioradialis and tibialis anterior muscles was performed at several insertion points and was used to screen for spontaneous activity, and, if possible, motor unit potential analysis was performed depending on patient cooperation. In addition, direct muscle stimulation and calculation of the ratio of the nerve and muscle evoked compound muscle action potentials were performed for the tibialis anterior muscle. For direct muscle stimulation, the stimulation cathode (insulated monopolar needle electrode: ECA, VIASYS Healthcare, Madison, Wisconsin, USA) was inserted perpendicularly into the tibialis anterior muscle distal to the motor endplate region. A non-polarisable surface electrode (Red Dot, 3 M Health Care, D-46325 Borken, Germany) served as anode and was placed approximately 2 cm lateral to the cathode. Current pulses of 0.1 ms were used for stimulation and the stimulation intensity was increased until supramaximal stimulation was achieved. Recordings were performed with a concentric 30G EMG electrode (Medtronic, Skovlunde, Denmark). This electrode was inserted into the tibialis anterior muscle approximately 15–30 mm proximal to the stimulation cathode. To calculate the ratio of the nerve and muscle action potentials, the peroneal nerve was stimulated distal to the fibular head and the maximum muscle action potential was measured with the same recording electrode as for direct muscle stimulation. For CIM diagnosis, the following electrophysiological criteria were considered relevant for diagnosis according to the diagnostic criteria (Latronico and Bolton, 2011): (1) compound muscle action potential amplitudes <80 % of the lower limit of normal in two motor nerves without signs of conduction block, (2) sensory nerve action potential amplitudes >80 % of the lower limit of normal, (3) EMG examinations with short-duration, low amplitude motor unit potentials with or without fibrillation potentials or, if motor potential analysis was not possible, reduced muscle fibre excitability on direct muscle stimulation, and (4) absence of a decremental response on repetitive nerve stimulation.

#### 2.2.3 Muscle biopsy

A muscle biopsy was performed on the tibialis anterior muscle using a 16 Gauge soft tissue semi-automated biopsy disposable needle instrument (Temno Evolution®) and immediately frozen at −80 °C. The determination of myosin:actin ratios followed previously established methods (Derde et al., 2012, Larsson and Moss, 1993). In brief, tissues were placed in lithium dodecyl sulfate buffer (pH 8.5) and subjected to polyacrylamide gel electrophoresis, followed by Coomassie staining. Gel imaging was performed using an iBright CL750 Imaging System (Thermo Fischer Scientific, MA, USA), and quantification of myosin and actin bands was carried out through densitometry analysis using ImageJ.

#### 2.2.4 Diagnosis of ICUAW and CIM

ICUAW was diagnosed if a patient met the following criteria (Schefold et al., 2020): (1) critical illness accompanied by multi-organ dysfunction and (2) limb weakness or challenges in ventilator weaning after ruling out non-neuromuscular factors with a MRC sum score of less than 48. CIM diagnosis was established if patients met the criteria for ICUAW (see above) and fulfilled in addition the following criteria (Latronico and Bolton, 2011): (3) electrophysiological criteria for CIM (see Section 2.2.2), and (4) preferential myosin loss in a muscle biopsy as outlined in the protocol by Marrero et al. (Marrero et al., 2020).

### 2.3 Statistical analysis

Statistical analyses were performed using SPSS Statistics 25.0 (IBM, Armonk, NY, United States). Patients were divided into groups twice: once into patients who developed ICUAW (ICUAW+) and those who did not (ICUAW−), and once into patients who developed CIM (CIM+) and those who did not (CIM−). The statistical analyses were based on these group classifications. Descriptive statistics were performed using frequencies and percentages for categorical variables and mean (±standard deviation) for continuous variables. Odds ratios (95 % confidence interval) were calculated for death in the ICU and at three months after ICU discharge for the outcomes of ICUAW and CIM. For the diagnosis of CIM, the sensitivity and specificity of each criterion was calculated separately. For all categorical criteria, sensitivity and specificity were calculated using crosstabs. The sensitivity and specificity of the myosin:actin ratio was assessed by a receiver operating characteristic (ROC) analysis.

## 3 Results

In this retrospective analysis of existing data from a prospective study, nine patients had to be excluded from the analyses because they were either still receiving neuromuscular blockers on the last examination day and therefore it was not possible to determine the MRC sum score and to perform extended electrophysiological examinations (n = 8), or the nerve conduction studies were incomplete (n = 1). The total sample size of the present study therefore is 22 patients. In this patient sample, the overall incidence of ICUAW was 92 % (20/22 patients) ten days after admission and the incidence of CIM was 55 % (12/22 patients; all of these patients also had ICUAW). Hence, 36 % (8/22 patients) fulfilled the criteria for ICUAW but did not develop CIM. According to the current diagnostic criteria, no patient developed critical illness polyneuropathy (Latronico and Bolton, 2011). Table 1 reports patient characteristics, length of ICU stay and frequency of death during ICU stay as well as at three months after ICU discharge. Overall, six (27 %) patients died during their ICU stay and 16 (73 %) survived. At three months after ICU discharge, six (27 %) patients had died, 13 (59 %) survived and in three patients (14 %) the outcome is unknown. All patients who died were among those who developed CIM: patients with ICUAW who developed CIM had a 50 % mortality rate both in the ICU and three months after discharge from the ICU, while no patient with ICUAW who did not develop CIM died. This is also reflected by the odds ratios for death, which were much higher for CIM than for ICUAW.

**Table 1:** Patient characteristics and outcomes.

	All (n = 22)	ICUAW− (n = 2)†	ICUAW+ (n = 20)†	OR (95 % CI)	CIM− (n = 10)†	CIM+ (n = 12)†	OR (95 % CI)	CIM− ICUAW+ (n = 8)†
Age	61.10 (±10.17)	75.00 (±5.00)	59.70 (±9.48)	−-	60.20 (±10.40)	61.83 (±10.81)	−-	56.50 (±6.86)
Sex (male)	17/22, 77 %	1/2, 50 %	16/20, 80 %	−-	6/10, 60 %	11/12, 92 %	−-	5/8, 63 %
ICU stay (days)	15.55 (±5.61)	6.50 (±1.50)	16.45 (±5.05)	−-	12.80 (±5.14)	17.83 (±5.37)	−-	14.38 (±4.09)
Death in ICU	6/22, 27 %	0/2, 0 %	6/20, 30 %	2.24 (0.09–53.59)	0/10, 0 %	6/12, 50 %	21 (1.01–438.25)	0/8, 0 %
Death at 3 months	6/22, 27 %	0/2, 0 %	6/20, 30 %	1.56 (0.06–43.94)	0/10, 0 %	6/12, 50 %	20.09 (0.93–432.78)	0/8, 0 %

Note. Data are reported as mean (±standard deviation) or absolute numbers (percentages). ICUAW, intensive care unit acquired weakness; OR, odds ratio; CI, confidence interval; CIM, critical illness myopathy; ICU, intensive care unit.

† The sample sizes of the different sub-groups refer to the total sample size of all included patients (n = 22).

The number and percentage of patients (overall and for each diagnostic sub-sample separately) meeting each of the diagnostic criteria for CIM are reported in Table 2. Of all patients, 92 % had clinical weakness and 55 % met the electrophysiological criteria for CIM. The mean myosin:actin ratio of all patients was 0.76 (±0.28). In patients who had ICUAW and developed CIM, the myosin:actin ratio was lower than in those who had ICUAW but did not develop CIM (0.66 (±0.19) vs. 0.93 (±0.35)). Both the clinical as well as electrophysiological criteria showed an excellent sensitivity for CIM diagnosis, but only the electrophysiological criteria had a high specificity (Table 3). The determination of the myosin:actin ratio showed a neither high sensitivity nor specificity for the diagnosis of CIM.

**Table 2:** Diagnostic criteria for CIM.

		All (n = 22)	ICUAW− (n = 2)†	ICUAW+ (n = 20)†	CIM− (n = 10)†	CIM+ (n = 12)†	CIM− ICUAW+ (n = 8)†
Clinical criteria	(1) Critical illness	22/22, 100 %	2/2, 100 %	20/20, 100 %	10/10, 100 %	12/12, 100 %	8/8, 100 %
	(2) Clinical weakness	20/22, 92 %	0/2, 0 %	20/20, 100 %	8/10, 80 %	12/12, 100 %	8/8, 100 %

Electrophysiological criteria	(3–6) Combined	12/22, 55 %	0/2, 0 %	12/20, 60 %	0/10, 0 %	12/12, 100 %	0/8, 0 %
	(3 & 4) NCS	12/22, 55 %	0/2, 0 %	12/20, 60 %	0/10, 0 %	12/12, 100 %	0/8, 0 %
	(5) EMG/Inexcitability	15/22, 68 %	0/2, 0 %	15/20, 75 %	3/10, 30 %	12/12, 100 %	3/8, 38 %
	(6) Repetitive Stimulation	0/22, 0 %	0/2, 0 %	0/20, 0 %	0/10, 0 %	0/12, 0 %	0/8,0 %

Muscle biopsy	(7) M:A ratio	0.76 (±0.28)	0.71 (±0.033)	0.77 (±0.29)	0.88 (±0.35)	0.66 (±0.19)	0.93 (±0.35)

Note. Data are reported as mean (±standard deviation) or absolute numbers (percentages). The diagnostic criteria for CIM are divided into clinical and electrophysiological criteria, and muscle biopsy. See Latronico and Bolton (2011) for a detailed description of the individual criteria. ICUAW, intensive care unit acquired weakness; CIM, critical illness myopathy; NCS, nerve conduction studies; EMG, electromyography; M:A ratio, myosin:actin ratio.

† The sample sizes of the different sub-groups refer to the total sample size of all included patients (n = 22).

**Table 3:** Sensitivity and specificity of the diagnostic criteria for CIM.

		CIM− (n = 10)	CIM+ (n = 12)	Sensitivity for CIM diagnosis	Specificity for CIM diagnosis
Clinical criteria	(1) Critical illness	10/10, 100 %	12/12, 100 %	−	−
	(2) Clinical weakness	8/10, 80 %	12/12, 100 %	100 %	20 %

electrophysiological criteria	(3–6) Combined	0/10, 0 %	12/12, 100 %	100 %	100 %
	(3 & 4) NCS	0/10, 0 %	12/12, 100 %	100 %	100 %
	(5) EMG/Inexcitability	3/10, 30 %	12/12, 100 %	100 %	70 %
	(6) Repetitive Stimulation	0/10, 0 %	0/12, 0 %	−	−

Muscle biopsy	(7) M:A ratio	0.88 (±0.35)	0.66 (±0.19)	33 %	50 %

Note. Data are reported as mean (±standard deviation) or absolute numbers (percentages). The diagnostic criteria for CIM are divided into clinical and electrophysiological criteria, and muscle biopsy. See Latronico and Bolton (2011) for a detailed description of the individual criteria. CIM, critical illness myopathy; NCS, nerve conduction studies; EMG, electromyography; M:A ratio, myosin:actin ratio.

## 4 Discussion

In this retrospective study, prospectively acquired data of critically ill COVID-19 patients with acute respiratory distress syndrome was analysed with regard to frequency and prognostic value of ICUAW and CIM. The diagnoses of ICUAW and CIM were established ten days after ICU admission using the current applicable diagnostic criteria set for these diseases (De Jonghe et al., 2002, Kleyweg et al., 1991, Latronico and Bolton, 2011, Schefold et al., 2020). In our patient cohort, 92 % of all patients fulfilled the criteria for ICUAW, whereas only 55 % met those for CIM. The diagnosis of CIM was more strongly associated with death during ICU stay than ICUAW. No patient with ICUAW who did not fulfil the criteria for CIM died.

Our results raise questions about the value and impact of the diagnosis of “ICUAW”, both clinically and scientifically. As many as 92 % of all patients in our cohort fulfilled the clinical criteria for ICUAW. The diagnosis of ICUAW relies on a purely clinical assessment of muscle force and entails some difficulties. The assessment of the MRC sum score, which is required for the diagnosis of ICUAW, depends on patient cooperation and is therefore not always reliable (e.g. necessity for analgo-sedation at the time of clinical examination, concomitant encephalopathy or delirium) resulting in an increased risk of a false-positive diagnosis of ICUAW. Furthermore, as mentioned in the introduction, ICUAW is an umbrella diagnosis that includes multiple pathologies, all of which have different underlying pathophysiologies, and as a consequence diverging mortality and recovery rates (Howard et al., 2008). This is also reflected in our results on mortality in the different patient groups: Overall, the mortality rate during the ICU stay and after three months was 27 %. All patients who died were among the patients that fulfilled the criteria for ICUAW and had developed CIM. In contrast, none of the patients who had ICUAW but did not develop CIM died. This suggest that patients who experience muscle weakness during their critical illness that was not caused by CIM had a much higher chance of survival. The odds ratios for death both in the ICU and in the first three months after discharge from the ICU were much higher for CIM than for ICUAW, with the latter including both patients with and without CIM. The development of CIM appears to be particularly associated with mortality during ICU stay. These findings further emphasize the greater prognostic value of a specific diagnosis such as CIM over the overarching diagnosis of ICUAW. Therefore, where possible, the diagnosis of ICUAW should not be used for research purposes, and in a clinical context only with caution and taking into account the limitations of this umbrella diagnosis, particularly with regard to predicting outcome. In order to develop effective preventive or therapeutic measures against weakness in critically ill patients, the aetiology and pathophysiology of muscle dysfunction must be known as well as possible. In homogenous patient groups with respect to the underlying pathology of muscle weakness, the pathophysiologies can be more accurately determined so that more specific treatments can be developed, and the effect of a treatment can be better assessed. The use of ICUAW as a diagnosis in treatment trials, which leads to a heterogeneous patient group with several different causes of muscle weakness, may therefore be a reason for negative or inconclusive treatment studies in the past.

The sensitivity and specificity analyses of the diagnostic criteria for CIM show that the current multimodal electrophysiological criteria consisting of sensory-motor nerve conduction studies, electromyographic evaluations and repetitive stimulation are superior to the clinical criteria alone in correctly diagnosing CIM. This finding is supported by a previous study stating that the incidence of failure of diagnostic assessment was much higher with a purely clinical approach (26 %) than with an electrophysiological technique (2 %) (Appleton et al., 2015). Unexpectedly, muscle tissue assessments measuring the myosin:actin ratio appear to have a lower accuracy for correctly diagnosing CIM by itself than electrophysiology. An incipient change or inexcitability of the muscle membrane does not necessarily have to be reflected by a structural change in the muscle. It has been shown that the muscle membrane can become inexcitable due to an acquired channelopathy of the voltage-gated sodium channels before structural changes occur in the muscle (Latronico and Friedrich, 2019, Z'Graggen et al., 2011). The utility of muscle biopsy in the diagnosis of CIM therefore needs to be further investigated and critically discussed. The current diagnostic criteria demand muscle tissue sampling (myosin:actin ratio or microscopic evaluation) for the diagnosis of “definite” CIM. Without that, only a “probable” diagnosis of CIM is possible. The invasiveness of a muscle biopsy and the delay in diagnosis due to the required time for processing and analysis of the muscle tissue sample are among the main reasons why clinicians and researchers rarely pursue the diagnostic recommendations for the evaluation of definite CIM and why the diagnosis of ICUAW is becoming more widely used. Hence, if the muscle biopsy could be omitted in the diagnostic process for CIM, the barrier to pursuing a specific diagnosis would probably be lowered.

There are several limitations to this study that need to be considered. First, the sample size is small, especially the number of patients who did not develop clinical weakness during the ICU stay. Due to this very small number, further statistical analyses including this sub-group were not feasible. Furthermore, as the outcome at three months was unknown in 14 % of patients, the actual mortality rate may have been higher than reported. We also did not assess the patients’ quality of life or degree of disability three months after ICU discharge, and thus no more precise conclusions can be made on the patients’ outcome. Lastly, as the study included only critically ill COVID-19 patients, the generalizability to critically ill non-COVID-19 patients is limited. The frequencies of ICUAW and CIM reported should be interpreted with caution, as COVID-19 itself may trigger myopathic processes (Abrams et al., 2023, Rahiminezhad et al., 2023). Due to these limitations, the results of the present study should be considered preliminary and need to be confirmed in a prospective study with a larger and more heterogeneous patient sample.

In conclusion, the results of the present study indicate that ICUAW is not a sufficiently specific diagnosis and is therefore of low clinical value in terms of outcome prediction. The mortality during ICU stay was higher among patients who had ICUAW with CIM compared to patients who had ICUAW but were not diagnosed with CIM. Thus, the diagnosis of CIM is more strongly associated with death during critical illness than the diagnosis of ICUAW. In addition, the diagnosis of ICUAW is also of little research value for exploring the aetiology and pathophysiology of muscle weakness in critically ill patients as well as for evaluating treatment efficacy. For these purposes, more specific diagnoses such as CIM seem more appropriate. Within the different diagnostic criteria for CIM, electrophysiological studies are the most sensitive and specific examinations compared to clinical and muscle tissue assessment.

## Competing interests

BR, JCS and WJZ report no competing interests.

## Author contributions

BR: conception and design of the study, analysis of data, and drafting a significant portion of the manuscript; JCS: conception and design of the study, and drafting a significant portion of the manuscript; WJZ: conception and design of the study, analysis of data, and drafting a significant portion of the manuscript.

## Funding

The study was supported by the 10.13039/501100001711Swiss National Science Foundation (grant number 32003B_215334) and grants of the 10.13039/100010767Swiss Foundation for Research on Muscle Diseases and the 10.13039/100009068University of Bern held by W.J. Z’Graggen.

## Data Availability

Anonymised data are available upon reasonable request to the corresponding author. Applications will be considered on an individual basis, assessing the feasibility and appropriateness of the proposed study and the ability to ensure the required level of data security in accordance with the original ethics committee. Data transfer will be regulated by a material transfer agreement.
